# Effects of Non-Invasive Neuromodulation of the Vagus Nerve for Management of Tinnitus: A Systematic Review with Meta-Analysis

**DOI:** 10.3390/jcm12113673

**Published:** 2023-05-25

**Authors:** David Fernández-Hernando, Cesar Fernández-de-las-Peñas, Ana Machado-Martín, Santiago Angulo-Díaz-Parreño, Francisco J. García-Esteo, Juan A. Mesa-Jiménez

**Affiliations:** 1 Universidad San Pablo-CEU, CEU Universities, Urbanización Montepríncipe, 28660 Boadilla del Monte, Spainjmesaj@ceu.es (J.A.M.-J.); 2 Department of Physical Therapy, Occupational Therapy, Physical Medicine and Rehabilitation, Universidad Rey Juan Carlos, 28922 Alcorcón, Spain; 3 Servicio de Otorrino-Laringología, Hospital Universitario Quiron Salud Pozuelo, Universidad Europea de Madrid, 28670 Madrid, Spain; 4 Facultad de Medicina, Universidad San Pablo CEU, 28660 Madrid, Spain

**Keywords:** meta-analysis, tinnitus, non-invasive neuromodulation, vagus nerve, related disability, auricular transcutaneous vagus nerve stimulation

## Abstract

Background: Tinnitus is the perception of sound in the absence of actual external stimuli. Other associated symptoms include frustration, annoyance, anxiety, depression, stress, cognitive dysfunction, insomnia, or emotional exhaustion. Objective: In this study, we aimed to conduct a systematic review and meta-analysis on the effectiveness of the non-invasive neuromodulation of the vagus nerve in patients with tinnitus. Methods: Six databases were searched from their date of inception to 15 June 2022 to identify clinical trials in which at least one group received any form of non-invasive neuromodulation of the vagus nerve for tinnitus management, with outcomes based on annoyance and related disability. Data on participants, interventions, blinding strategies, assessment outcomes, and results were extracted by two reviewers. Results: The search identified 183 articles with five clinical trials eligible for inclusion in the review and four for the meta-analysis. The methodological quality scores ranged from 6 to 8 (mean: 7.3, SD: 0.8) points. The meta-analysis identified a significant positive effect on THI post-treatment for unilateral auricular stimulation (hg = 0.69, 95% CI 0.06, 1.32) or transcutaneous nerve stimulation (hg = 0.51, 95% CI 0.1, 0.9) compared with a comparative group. No effect on loudness intensity was observed. Conclusion: The results of the meta-analysis suggest that the application of the non-invasive neuromodulation of the vagus nerve has a positive effect post-treatment in terms of related disability in patients with tinnitus, although its clinical relevance is low. No firm conclusions about the effect of the non-invasive neuromodulation of the vagus nerve on tinnitus are available based on the current literature.

## 1. Introduction

Auricular transcutaneous vagus nerve stimulation (at-VNS) is a modality of non-invasive nerve stimulation (n-VNS), commonly used in clinical practice to treat tinnitus (acute or chronic), cluster headaches, migraines, depression, epilepsy, and other disorders, as well as atrial fibrillation, prosocial behavior, associative memory, schizophrenia, or pain [1,2,3,4]. n-VNS is a safe, reliable treatment with no adverse effects in response to medication intake [4,5]. At-VNS is a non-invasive and non-expensive therapy that involves stimulating the auricular branch of the vagus nerve (ABVN) at the outer parts of the ear, conferring autonomic benefits [6,7,8]. One of the main differences between VNS and at-VNS is that, in at-VNS, patients do not need general anesthesia for implantation, making this treatment safer than VNS [3,4]. The former method is also more expensive and riskier than at-VNS, with costs ranging from USD 30,000 to USD 50,000 [5,9]. Magnetic resonance imaging (MRI) has recently highlighted an anatomical pathway for at-VNS, for example, through the neck and auricula, which connect the nervous system and the external parts of the body [7,8,10]. The physiological effects of at-VNS on the brain have yet to be fully elucidated, and studies are not homogeneous in their results due to the high risk of bias and unclear parameters, stimulation intensity, pulse width, waveform, or frequency. 

Tinnitus is the perception of sound in the absence of actual external sounds. It has an annual prevalence of 1%, being experienced by 14% of the population. Tinnitus is not a severe otological symptom in most cases, and only 2% of cases are severe [11,12,13,14]. Its prevalence does not differ by sex but increases with age, affecting 10% of young adults, 14% of middle-aged people, and 24% of adults as a whole [11,14]. Symptoms include annoyance, anxiety, depression, cognitive dysfunction, insomnia, and emotional exhaustion. Patients with tinnitus experience hearing loss, with ipsilateral tinnitus on one side being the most reported pathology [11,12,15]. A possible explanation for tinnitus is that the damage to the inner ear involving hair cells causes hearing loss, and the input enters the cochlea, with the latter sending incorrect information to the superior level on the brain cortex—more specifically, the auditory cortex. Physical tests such as audiograms may be normal in these patients [10,11,12,15,16].

Recently, a systematic review on the treatment of tinnitus with n-VNS including some studies with invasive treatment was published, differing completely from this systematic review, which only includes non-invasive neuromodulation; however, no meta-analysis was conducted, and it included only two randomized controlled trials (RCTs), five cohorts studies, and two case series [17]. Meanwhile, this systematic review and meta-analysis included randomized controlled trials (RCTs) as well as controlled trials that related the quality of studies and the effectivity of n-VNS as a treatment for tinnitus with a novel technique, at-VNS, distinguishing them completely from all the RCTs included in both systematic reviews. Furthermore, a recent study provides evidence applied in rats that VNS is more useful and safer in combination with drugs to prevent adverse events and that VNS therapy mediates plasticity and enhances recovery as part of neuro-rehabilitation [18]. 

## 2. Materials and Methods

A systematic review and meta-analysis following the Preferred Reporting Items for Systematic Reviews and Meta-Analyses (PRISMA) guidelines was conducted [19]. This review was prospectively registered in the PROSPERO database (registration number: CRD42021265126).

### 2.1. Search Strategy

Electronic literature searches were conducted on the CINALH, MEDLINE, SCOPUS, PUB MED, PEDro, and EMBASE databases from their inception to 15 June 2022. When the searched databases allowed limits, searches were restricted to randomized clinical trials. We also screened the reference lists of the papers identified in the searches. The search strategy included the following MeSH terms: “Vagus Nerve”, “Auricular Vagus Nerve Stimulation”, “Transcutaneous Vagus Nerve Stimulation”, and “Tinnitus”. These terms were combined with “AND” or “OR” operators, as follows:

((Non-Invasive neuromodulation) OR (auricular Transcutaneous Vagus Nerve Stimulation) OR (Transcutaneous Vagus Nerve Stimulation)) AND ((Tinnitus) OR (Chronic Tinnitus)).

### 2.2. PICO Principle

This review/meta-analysis was conducted to answer the following clinical question: Is it effective to use at-VNS to treat tinnitus? 

Population, Intervention, Comparison, and Outcome (PICO) questions. 

Population: Adults older than 18 years of age with chronic tinnitus or severe tinnitus. 

Intervention: Application of auricular transcutaneous vagus nerve stimulation technique. 

Comparator: Acceptable comparators were any type of placebo (e.g., turning off device), sham, or no intervention. 

Outcomes: The primary outcome measure was tinnitus annoyance (assessed by a visual analogue scale, VAS, or numerical scale), related disability (assessed by the Tinnitus Handicap index, THI), or hearing loss (audiometry, pure tone average).

### 2.3. Study Selection

Inclusion criteria: (i) Population: studies with patients of any age or sex with a chronic or severe tinnitus diagnosis made by a health professional. (ii) Application of auricular transcutaneous vagus nerve stimulation technique. (iii) Comparison: Acceptable comparators were any type of placebo (e.g., turning off device), sham, or no intervention. (iv) Outcome: The primary outcome measure was tinnitus annoyance (assessed by a visual analogue scale, VAS, or numerical scale), related disability (assessed by the Tinnitus Handicap Index, THI), or hearing loss (audiometry, pure tone average). (v) Studies: This review included randomized controlled, clinical trials, controlled trials, and pilot clinical trials since these designs were suitable to answer our question.

Exclusion criteria: (i) Population: studies whose patients had no evidence of any tinnitus diagnosis. (ii) Intervention/exposure/factor: studies that used surgical interventions of VNS. (iii) Comparison: studies with no comparison group. (iv) Studies: systematic reviews, meta-analyses, reviews, case reports, letters to the editor, conference articles, book chapters, protocol registries, grey literature, cadaver studies, and animal studies.

### 2.4. Data Extraction and Quality Assessment

Data extraction was performed by two authors, and the data were compiled into a standardized data extraction form in an excel spreadsheet. Data included simple size, diagnosis, inclusion/exclusion criteria, duration of symptoms, intervention (location, technique, and duration), main outcomes, follow-ups, and adverse events. In case of a discrepancy between authors, an agreement had to be achieved; a third author was in charge of reaching a consensus.

Methodological quality was evaluated using the PEDro (Physiotherapy Evidence Database) scale and the ROB-2 Cochrane tool; this process was carried out independently by two authors. The RoB-2 tool includes the following items: selection bias (randomization sequence generation, allocation concealment), performance bias (blinding participants, blinding therapists), detection bias (blinding outcome assessor), attrition bias (incomplete outcome data), reporting bias (source of funding bias/selecting outcome reporting), and other bias (sample size) [20]. Each item was classified as low risk, high risk, or unclear, according to the Cochrane Collaboration tool [20]. In all cases, an answer ‘Yes’ indicated a low risk of bias, and an answer ‘No’ indicated a high risk of bias. If insufficient details were reported regarding what occurred during the trial or the entry was not relevant to the study (particularly for assessing blinding and incomplete outcome data, when the outcome being assessed by the entry had not been measured in the study) the answer was ‘unclear’ risk of bias [20,21].

The PEDro scale is based on 11 criteria, of which 10 contribute to the score, representing methodological quality. The PEDro scale has been shown to have fair to good interrater reliability (ICC 0.55, 95% CI 0.41–0.72) [22]. The scale assessed the following items: random allocation; concealed allocation; between-groups similarity at baseline; participant blinding; therapist blinding; assessor blinding; dropout; intention-to-treat statistical analysis; between-group statistical comparison; point measures; and variability data. A PEDro score ≥ 5/10 points determines a high-quality trial. 

### 2.5. Level of Evidence

We used the Grading of Recommendations Assessment, Development, and Evaluation (GRADE) approach [23]. According to the GRADE approach, the level of evidence can be classified as high quality (the authors are very confident that the intervention effect is close to the estimated effect), moderate quality (the authors are somewhat confident that the intervention effect is probably close to the estimated effect, but there is a possibility that it is different), and low quality (the intervention effect may be markedly different from the estimated effect) [23]. GRADE assesses quality according to four main domains: risk of bias, inconsistency, indirectness, and imprecision. The overall classification of the evidence is assessed as either “high”, “moderate”, “low”, or “very low”. For risk of bias, we established that a “very low” quality rating required a study to receive a ≥2 high risk of bias rating or 1 specific high risk rating in the “other category”, plus a >2 unclear rating or 3 unclear rating in dimensions other than the “other category” using the Cochrane risk of bias tool. A “low” quality rating required that a study receive at least 1 high risk rating; a “moderate” quality rating required that the study received 2 unclear risk of bias ratings in a category related to randomization and/or blinding; and a “high” quality rating required that a study receive 1 or fewer unclear risks of bias. 

### 2.6. Data Synthesis and Analysis

We extracted the sample size, means, and standard deviations for each variable. When the trial reported standard error or non-parametric values (median or interquartile range), they were converted to standard deviations and parametric values as needed. If necessary, the mean scores and standard deviations were estimated from graphs. 

The between-groups mean differences of the trials were converted to effect sizes using a random-effects model. An effect size of 0.8 or greater was considered large, between 0.5 and 0.8 was considered moderate, and between 0.2 and 0.5 was considered small. *p*-values < 0.05 were considered statistically significant. Due to the small number of studies identified in the review, the meta-analytic comparison was based on the mean difference change (hg) in the tinnitus variable (THI, VAS loudness, or VAS uncomfortable) at post-treatment and in the short term (four-weeks) after at-VNS compared with the control or comparative group.

For inconsistency, we used I^2^ heterogeneity between studies, estimated using Cochrane. The Cochrane group establishes the following interpretation of the I^2^ statistic: 0–40% may represent irrelevant/unimportant heterogeneity, 30–60% suggests moderate heterogeneity, 50–90% represents substantial heterogeneity, and 75–100% represents considerable heterogeneity [24]. As described by the Cochrane group, the data of heterogeneity overlap. The importance of the observed value of I^2^ is based on the magnitude and the direction of effects and the strength of evidence of heterogeneity (*p* value). In grading the imprecision of continuous outcomes, the effect size magnitude cutoffs proposed by Cohen were used as benchmarks for assessing the magnitude of the confidence intervals of observed effects. For example, 95% confidence intervals (CI) of a standardized mean difference (SMD) extending between >0.2 and <0.5 points in either direction indicate a “serious” quality downgrade; 95% CI of an SMD extending >0.5 point in either direction indicate a “very serious” quality downgrade [25].

Publication bias was assessed graphically using funnel plots and the Egger regression test [23]. Statistical assessment was two-tailed and was considered statistically significant at *p* < 0.05 [25,26,27]. The sensitivity analysis was performed using MIX 2.0 Pro.

## 3. Results

### 3.1. Study Selection

**Figure 1:**
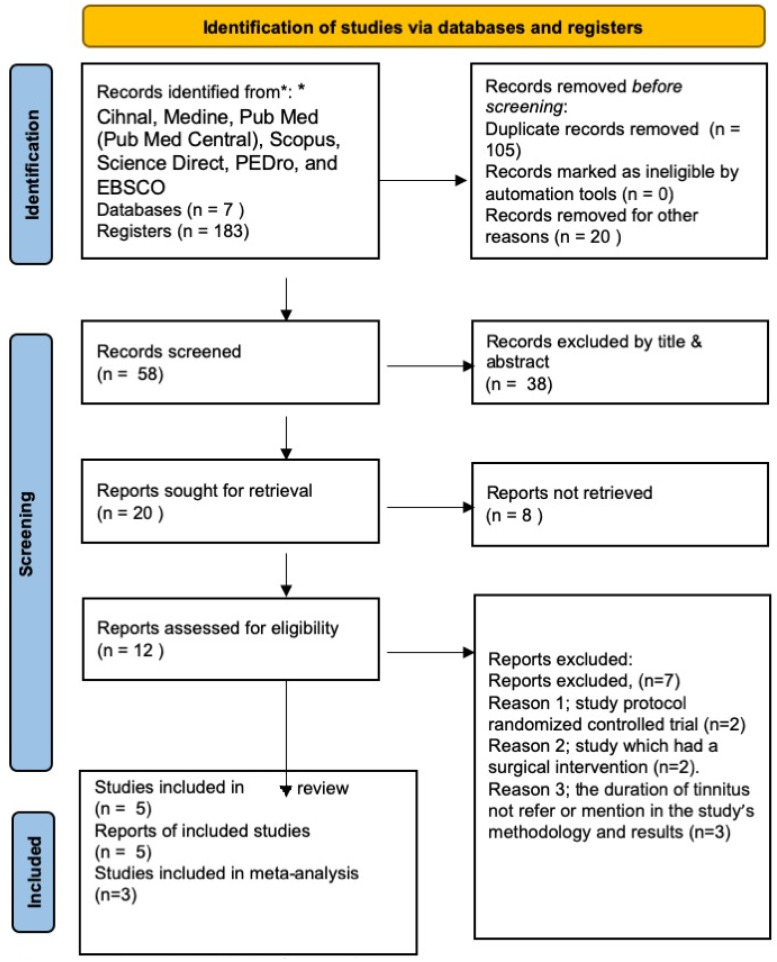
PRISMA flow diagram.

The data search yielded a total of 183 articles, including duplicates. Thirty-eight articles were included for abstract/full text review, five were included in the systematic review [28,29,30,31,32], and four were included in the meta-analysis [29,30,31], while three were excluded based on the inclusion and exclusion criteria [33,34,35]. Figure 1 shows the flow diagram of the data search and the studies that met the inclusion/exclusion criteria.

### 3.2. Study Characteristics

The total sample size consisted of 234 participants (age: 47, 93, SD: 11, 49 years, 61.96% men). The duration of tinnitus-associated symptoms ranged from 3 months to 2 years (mean: 51.3, SD: 58 weeks). The article types included three RCTs [29,30,31], one clinical controlled trial [32], and one clinical trial [28], and there were three excluded studies, which did not report the tinnitus duration or hearing loss threshold or the article type [33,34,35] (Table 1). All the studies included in this systematic review at baseline characteristics measured tinnitus duration and audiological assessment of hearing loss threshold (pure tone audiometry, tympanometry, speech discrimination score).

### 3.3. Outcomes and Follow-Ups

We extracted the following outcomes from the THI [29,30,31,32] and VAS: Loudness, annoyance, or discomfort [29,30,31] were the most commonly assessed, while others included pure tone average (PTA), speech discrimination [28,29,31,32], depression anxiety stress scales (DASS) [29], audiometry [28,29,30,31,32], MRI for cortical activation [32], heart rate, vagal somatosensory evoked potential test, minimum masking level (MML), tinnitus loudness matching (TLM), audiograms, and pure tone thresholds [29].

Five studies evaluated outcomes immediately after the last treatment session [28,29,31,32,35], whereas two trials evaluated the effects in the short term (4 weeks after) [30,31].

### 3.4. Interventions/Control Groups

For the intervention group, it is important to underline the types of modalities including at-VNS. Some studies used a turn-on Transcutaneous Electrical Nerve Stimulation (TENS) in the auricular branch of the vagus nerve [29,30,31,32]. Another type of intervention used monomodal versus bi-modal somatosensory stimulation [28].

The parameters of electrical stimulation were inconsistent in frequency, interval time, or treatment time among trials. While there were four trials with similar parameters in terms of current frequency (i.e., 20–30 Hz) [29], the pulse width, treatment, and electrodes placement were consistently different. If the area of application was similar, the position did not remain the same, even if it depended on the type of intervention or study protocol. The most common areas for electrode placement were the inner tragus, cymba conchae, C2 dermatome, triangular fossa, inner ear, meatus, and tragus [29,30,31,32].

The control group in the studies exhibited the following factors: turn-off devices for sham at-VNS [29,32]; anatomical regions other than the ear lobe combined with manual acupuncture [30,31]; another, different intervention [28].

### 3.5. Methodological Quality and Risk of Bias

**Figure 2:**
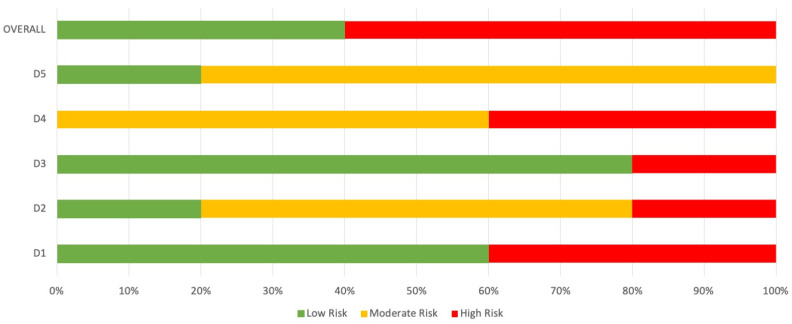
Rob-2 Tool Cochrane. D1. Randomization process; D2. deviations from the intended interventions; D3. missing outcome data; D4. measurement of the outcome; D5. selection of the reported results.

The methodological quality scores ranged from 6 to 8 (mean: 7.3, SD: 0.8) out of a maximum of 10 points (Table 2). Only three studies were of high methodological quality (≥5 points) [29,30,31], as shown in Figure 2. The ROB-2 Cochrane tool demonstrated that all studies had high risk of bias in items, except for two RCTs that had low risk [30,31]. The others all had high risk [28,29,32]. The overall results show us 40% of low risk and 60% of high risk of bias in the assessment. For each domain the assessment was D1; 60% for low risk and 40% for high risk of bias, D2; 20% for low and high risk, respectively, and 60% for moderate risk of bias, D3; 80% for low risk and 20% for high risk of bias, D4; 60% for moderate and 40% for high risk of bias, and D5; 20% for low risk and 80% for moderate risk of bias.

### 3.6. Unilateral Auricular Stimulation vs. Control

**Figure 3:**
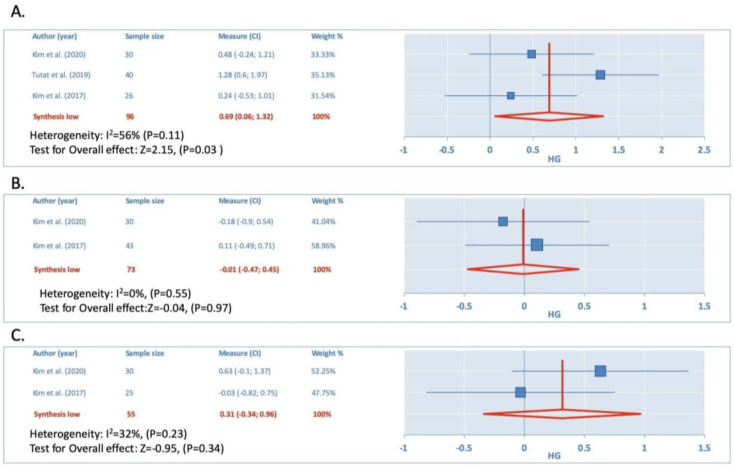
(**A**) Comparison of the effects of unilateral auricular stimulation vs. Control for THI. (**B**) Comparison of the effects of unilateral auricular stimulation vs. Control for VAS Loudness. (**C**) Comparison of the effects of unilateral auricular stimulation vs. Control for VAS Uncomfortable [29,30,31].

The meta-analysis revealed a significant effect on THI (hg = 0.69, 95% CI 0.06 to 1.32, Z = 2.15, *p* = 0.04, I^2^ = 56%, n = 3, Figure 3A) but not on VAS loudness (hg −0.01, 95% CI −0.47 to 0.45, Z = −0.04, *p* = 0.97, I^2^ = 0%, n = 2, Figure 3B) or VAS uncomfortable (hg = 0.31, 95% CI −0.34 to 0.96; Z = 0.95, *p* = 0.34, I^2^ = 32%, n = 2, Figure 3C) post-treatment. 

No significant effect on THI (hg = 0.28, 95% CI −0.25 to 0.81; Z = 1,03, *p* = 0.3, I^2^ = 0%, n = 2), VAS loudness (hg = −0.02, 95% CI −0.48 to 0.44; Z = −0.1, *p* = 0.92, I^2^ = 0%, n = 2), or VAS uncomfortable (hg = 0.31, 95% CI −0.16 to 0.77; Z = 1.29; *p* = 0.2; I^2^ = 0%, n = 2) at short-term was found either.

### 3.7. Unilateral Auricular Stimulation vs. Bilateral Stimulation

The meta-analysis revealed a non-significant effect on THI (hg = 0.21, 95% CI −0.13 to 0.55; Z = 1.21, *p* = 0.23, I^2^ = 0%, n = 4), VAS loudness (hg = 0.19, 95% CI −0.34 to 0.71, Z = 0.7, *p* = 0.49, I^2^ = 0%, n = 2), and VAS uncomfortable (hg = 0.15, 95% CI −0.38 to 0.67, Z = 0.55, *p* = 0.58, I^2^ = 0%, n = 2) post-treatment. There was no significant effect on THI (hg = 0.3, 95% CI −0.22 to 0.83; Z = 1.13, *p* = 0.26, I^2^ = 0%, n = 2), VAS loudness (hg = 0.13, 95% CI −0.39 to 0.65, Z = 0.48, *p* = 0.63, I^2^ = 0%n = 2), or VAS uncomfortable (hg = 0.16, 95% CI −0.36 to 0.67, Z = 0.59, *p* = 0.55, I^2^ = 0%, n = 2) in the short term.

### 3.8. TENS vs. Control

The meta-analysis revealed a significant effect on THI (hg = 0.51, 95% CI 0.1 to 0.9, Z = 2.45, *p* = 0.01, I^2^ = 0%, n = 3), but not on VAS loudness (hg = 0.19, 95% CI −0.34 to 0.71, Z = 0.7, *p* = 0.49, I^2^ = 0%, n = 2) or on VAS uncomfortable (hg = 0.07, 95% CI −0.45 to 0.59, Z = 0.27, *p* = 0.79, I^2^ = 0%, n = 2) post-treatment. No significant effects on THI (hg = 0.3, 95% CI −0.22 to 0.83, Z = 1.14, *p* = 0.26, I^2^ = 0%), VAS loudness (hg = 0.13, 95% CI −0.39 to 0.65, Z = 0.48, *p* = 0.63, I^2^ = 0%, n = 2), or VAS uncomfortable (hg = 0.16, 95% CI −0.36 to 0.68, Z = 0.6, *p* = 0.55, I^2^ = 0%, n = 2) were observed in the short term. Quality of Evidence (GRADE).

The low quality of evidence (heterogeneity) must be taken carefully because of the small number of studies available and the low quality of some of them in terms of the effectiveness of non-invasive neuromodulation for tinnitus in the short and long term (Table 3). 

## 4. Discussion

The objective of this meta-analysis was to determine the effects of non-invasive neuromodulation of the vagus nerve for the management of tinnitus-associated symptoms. The results suggest that the application of non-invasive neuromodulation of the vagus nerve has a positive post-treatment effect on related disability in patients with tinnitus, although its clinical relevance is small. 

Certain points are in need of further consideration, as a recent systematic review and an n-NVS-related review published in recent years [17] both revealed slightly different findings. The first difference was in the eligibility criteria, since we only included clinical trials, whereas Stegeman et al. [17] included case reports, case series, cohorts studies, and only two RCTs. The inclusion of just clinical trials allowed us to pool data and conduct the first meta-analysis. Secondly, the assessment of methodological quality or risk of bias revealed a high risk of bias and several concerns regarding the quality of trials [28,29,32,33,34,35]. These studies also have a control group which can be used to compare the interventions with turn-off devices or different interventions or to compare the same intervention involving different anatomical spaces [28,29,30,31,32,33,34,35]. In the last publication, there were no comparisons, indicating that there is a high risk of bias regarding conclusions [17], with one exception being the RCT crossover study that was excluded from our systematic review because it had an invasive intervention [17,36]. Additionally, another important difference was that the last publication divided into VNS with paired sound and VNS without paired sound including a unique study where at-VNS was used, but that was a cohort prospective study [17].

Recently, VNS in rats was proven to be a remarkable neuro-rehabilitation therapy because it influences network connectivity and motor control. It releases pro-plasticity factors such as norepinephrine, acetylcholine, serotonin, brain-derived neurotrophic factor, and fibroblast growth factor [37]. The majority of them were identified in spinal cord injuries or brain injuries in rat models. However, these studies demonstrate the pathways and how neuroplasticity works. VNS therapy can stimulate muscles, nerves, and the brain; thus, it could help to treat pathologies such as spinal cord injuries because it strengthens the connectivity between them and assists with recovery. Furthermore, VNS therapy takes more evidence in the activation of cholinergic and noradrenergic modulatory pathways [38,39,40]. Furthermore, these new advances could have application in humans as a new treatment for spinal cord injuries or any neurological disorders such as tinnitus. 

In the last year, findings have emerged which support the new advances in neuromodulation to treat neurological disorders. These techniques with an implant demonstrate the relationship between peripheral nerves and the central nervous system. It is important to underline that they are their safety and reliability as a neuromodulation treatment has not yet been proven. They are more accurate than other treatments in terms of the specific parameters, with greater efficacy [39]. There are also non-invasive devices which represent a new clinical treatment option, as demonstrated in recent clinical trials; these devices induce tactile stimulation of the Vagus nerve using a VNS implantation. This technique has provided evidence of enhanced peripheral stimulation, suggesting that neuroplasticity can be used to recover motor functions after brain damage [41]. 

This study also identified heterogeneity in the parameters of the interventions in terms of frequency, intensity, and wave pulse, which made it difficult to determine which one was effective. Simultaneously, the number of sessions, position of electrodes, and duration of treatment were also different, and a consensus is therefore necessary. Further, randomized clinical trials must be contemporary to guarantee their methodological quality [42,43,44,45]. For example, the latest publications that explore using acupuncture alone to treat tinnitus have the same results after the operation and three-weeks postoperatively, demonstrating that follow-up for this pathology has to be applied effectively to determine the presence of a positive or long-term effect [46].

Few trials have investigated the pathways of tinnitus, migraine, and epilepsy and the underlying mechanisms of how non-invasive neuromodulation of the vagus nerve could work [3,13,47]. This peripheral stimulation of the nervous system tries to change the central nervous system without adverse events, thus increasing the chances of successful treatment. Trying to understand the main pathways and mechanisms of action with which to treat these types of pathologies is a topic for further research. Additionally, not all studies followed the same protocol with the same parameters of the point of application of at-NVS, or ABVNS, based on this principle, which may be a different path of treatment. 

The follow-ups for measurements are inconsistent in all studies compared with the last publication [17]. Moreover, the results are the same immediately post-treatment [28,29,30,31,32,33,34,35] and four weeks later [30,31]. The lack of long-term follow-up periods makes it harder to draw a conclusion. 

In the meta-analysis, we have to make a strong critique regarding the low quality and the small number of studies because there were only three RCTs of high methodological quality (PEDro score > 5 points). The data provided were not the most suitable for comparison due to their heterogeneity [29,30,31], and most studies presented some concerns in relation to ROB-2 in the final assessment [32]. Even if the results are slightly positive for interventions, it is the same before intervention, and low THI increases the risk of bias and affects quality. For those reasons, we cannot draw any firm conclusion about the true effectiveness of at-VNS for the management of tinnitus. Moreover, it is important to underline the relation between hearing loss threshold and audiological examination at baseline and the results of THI since it is the main symptom of tinnitus and has shown the most improvement after treatment using at-VNS compared with control groups. Additionally, because patients with tinnitus have this chronic otological symptom related to hearing loss, they are classified as having severe or chronic tinnitus based on the duration of symptoms and age extracted from all studies included in this systematic review.Furthermore, slight improvement in hearing loss could have positive effects on other symptoms such as anxiety and the quality of life or life style of many suffering patients. Because of the previous critiques the improvement of the methodological study design is mandatory, and should incorporate the use of similar follow-ups and parameters to be able to determinate if at-VNS can improve this symptom based on the hearing loss threshold examination and consequently improve the quality of life of patients who suffer from chronic or severe tinnitus.

One of the main limitations of this meta-analysis is the small number of studies available and their poor methodological quality [29,30,31]. Most outcomes were no different for the intervention at any follow-up period. There were differences only in THI post-treatment, with some positive results in the TENS and unilateral auricular stimulation groups. Consequently, it is mandatory to provide RCTs with a better methodology and low risk of bias to provide strong evidence of the possible effectiveness of at-NVS in patients with tinnitus. Another limitation was the difference between parameters, the position of the electrodes, and protocols of the studies of at-VNS [30,31,32,33] and at-VNS [29,30,31,32,33,35] or bi-phasic stimulation [28], which made it difficult to verify which one is more effective for treating tinnitus.

## 5. Conclusions

The current meta-analysis on at-VNS found that non-invasive neuromodulation of the vagus nerve could have a slightly positive post-treatment effect on THI outcomes in patients with tinnitus, but its clinical relevance is small because of the number of studies available for the meta-analysis. The number of trials was small, and the studies presented high risk of bias. No conclusion about the effect of non-invasive neuromodulation of the vagus nerve on tinnitus is available based on the current literature. Of these studies, there were none of sufficient quality to asseverate the findings, and high-quality studies must be conducted in the future.

## Figures and Tables

**Table 1:** Summary of all included studies.

Study (Design)	Number of Participants & Male/Female	Age (Years)	Duration Tinnitus	Intervention Duration (Sessions/Weeks) Area of Treatment	Comparison and Outcome Measure	Between−Groups Differences (95% CI) [hg]
Kim et al., 2020 [30] (RCT)	G1; at-VNS; 15. G2; MA; 15 G3; 15 13/2 12/3 8/7	47.3 ± 9.5 48.6 ± 7.8 46.2 ± 11.0	98.1 ± 97.7 months 105.1 ± 142.0 months 107.8 ± 115.9 months	G1; 15; 30-Hz G2; MA G3; 30/90 Hz with a 3-second interval) 20 min for 10 sessions Inner tragus, cymba conchae	THI, VAS loudness, and VAS annoyance PTA, and speech discrimintion test G1 vs. G2 vs. G3	THI Post−treatment (−0.16, 1.3) [0.57] Short term (−0.25, 1.2) [0.48] VAS Loudness Post−treatment (−032, 1.13) [0.41] Short term (−0.53, 0.9) [0.18] VAS Uncomfortable Post−treatment (−0.45, 0.59) [0.07] Short term (−0.36, 0.68) [0.16]
Tutar et al., 2019 [29] (RCT)	G1; TENS (unilateral tinnitus); 20 G2; TENS (bilateral tinnitus); 20 G3; Control; 20 NR NR NR	41.17 ± 10.75	31 ± 49 months [range: 3–198 months]	G1 and G2; 1–200 Hz, 10–30 mA, 1000 msec 10 sessions in 1 month for 30 min (maximum of 4 days between each session) Inner tragus, cymba conchae	THI DASS (pure tone audiometry, tympanometry, speech discrimination score G1 vs. G2 vs. G3	THI Post−treatment (0.11, 1.4) [0.76]
Yakunina et al., 2018 [32] (CT)	G1; at-VNS with Tinnitus subjects; 36 G2; at-VNS with Normal subjects.; 37 27/9 18/19	51.0 ± 11.9 30.9 ± 8.2	63.2 ± 59.5 (3–134) months	G1 & G2; 500 μs, 25 Hz, 0.1 mA Unique session of MRI scan, total of six 5 min fMRI runs Inner tragus, cymba conchae	Hearing and tinnitus assessment MRI	NR
Kim et al., 2017 [31] (RCT)	G1; at-VNS (auricular region); 14. G2; MA; 13 G3; at-VNS (distal region), 15 12/2 6/6 10/3	54.6 ± 14.3 49.3 ± 15.6 53.9 ± 13.2	9.55 ± 11.55 years 7.1 ± 7.85 years 5.7 ± 6.4 years	G1 and G3; 4/100 Hz interval 3 s 20 min for 8 sessions 2 sessions/week Inner tragus, cymba conchae	THIS, VAS loudness, and VAS uncomfortable, PTA, and speech discrimintion test G1 vs. G2 vs. G3G1 vs. G2 Function (IKDC function subscale) G1 vs. G2 G1 vs. G2	THI Post−treatment (−0.66, 0.85) [0.1] Short Term (−0.64, 0.87) [0.12] VAS Loudness Post−treatment (−0.81, 0.7) [−0.06] Short term (−0.69, 0.82) [0.06] VAS Uncomfortable Post−treatment (−0.88, 0.63) [0.25] Short term (−0.69, 0.82) [0.06]
Hamilton et al., 2016 [28] (CT)	G1; Bi-modal stimulation Tinnitus compliant; NR G2; Bi-modal stimulation Tinnitus non-compliant; NR 34 (63%) men	47.5 ± 11	>2 years/<2 years 36 (78%)/10 (22%)	G1; Auditory stimulus G2; Bi-modal stimulation. 20 Hz—20 kHz For 14 weeks every visit per 2 weeks, 7 sessions, 47 min each session Inner tragus, cymba conchae	THI, MML, TLM, and VAS loudness G1 vs. G2	THI Post−treatment (−0.35, 0.93) [0.29]

DASS—Depression Anxiety Stress scale; THI—Tinnitus Handicap Index; TENS—transcutaneous electrical nerve stimulation; at-NVS—auricular transcutaneous Nerve Vagus stimulation; ABVN—auricular branch of the vagus nerve; MA—manual acupuncture; EA—electrical acupuncture; PTA—pure tone average;. SD—speech discrimination; HRV—heart rate variability; PE—periauricular electroacupuncture group; DE—distal electroacupuncture group; MML—minimum masking level; TLM—Tinnitus loudness matching; MEG—magnetoencephalography; fMRI—functional Magnetic Resonance Image; CT—clinical trial; RCT—randomized controlled trial.

**Table 2:** Score of randomized clinical trials with PEDro scale.

	1	2	3	4	5	6	7	8	9	10	Total
Tinnitus											
Kim et al., 2020 [30]	Y	Y	Y	N	N	N	N	Y	Y	Y	6/10
Tutar et al., 2019 [29]	Y	N	Y	N	N	Y	Y	N	Y	Y	6/10
Kim et al., 2017 [31]	Y	Y	Y	N	N	N	Y	Y	Y	Y	7/10
Yakunina et al., 2018 [32]	N	N	N	N	N	N	N	N	Y	Y	2/10
Hamilton et al., 2016 [28]	N	N	N	N	N	N	N	N	Y	Y	2/10

1: Random Allocation of Participants; 2: Concealed Allocation; 3: Similarity Between Groups at Baseline; 4: Participant Blinding; 5: Therapist Blinding; 6: Assessor Blinding; 7: Fewer than 15% Dropouts; 8: Intention- to-Treat Analysis; 9: Between-Group Statistical Comparisons; 10: Point Measures and Variability Data.

**Table 3:** The Grading of Recommendations Assessment, Development, and Evaluation (GRADE) Evidence Profile.

Number of Studies	Risk of Bias	Inconsistency	Indirectness of Evidence	Imprecision	Publication Bias	Quality of Evidence	hg [95% CI]
Unilateral auricular stimulation vs. Control THI							
Post-treatment group (n = 3)	Serious	High	Low/acceptable	No	Very serious	Very Low	0.69 (0.06, 1.32)
Short-term subgroup (n = 2)	No	Low	Low/acceptable	No	Very serious	Moderate	0.28 (−0.25, 0.81)
Unilateral auricular stimulation vs. Control Vas Loudness							
Post-treatment group (n = 2)	No	Low	Low/acceptable	No	Very serious	Moderate	−0.01 (−0.47, 0.45)
Short-term subgroup (n = 2)	No	Low	Low/acceptable	No	Very serious	Moderate	−0.02 (−0.48, 0.44)
Unilateral auricular stimulation vs. Control Vas Uncomfortable							
Post-treatment group (n = 2)	No	Low	Low/acceptable	No	Very serious	Moderate	0.31 (−0.34, 0.96)
Short-term subgroup (n = 2)	No	Low	Low/acceptable	No	Very serious	Moderate	0.31 (−0.16, 0.77)
Unilateral Stimulations vs. Bilateral THI							
Post-treatment group (n = 4)	Serious	High	Low/acceptable	No	Very serious	Very Low	0.21 (−0.13, 0.55)
Short-term subgroup (n = 2)	No	Low	Low/acceptable	No	Very serious	Moderate	0.3 (−0.22, 0.83)
Unilateral Stimulations vs. Bilateral Vas Loudness							
Post-treatment group (n = 2)	No	Low	Low/acceptable	No	Very serious	Moderate	0.19 (−0.34, 0.71)
Short-term subgroup (n = 2)	No	Low	Low/acceptable	No	Very serious	Moderate	0.13 (−0.39, 0.65)
Unilateral Stimulations vs. Bilateral Vas Uncomfortable							
Post-treatment group (n = 2)	No	Low	Low/acceptable	No	Very serious	Moderate	0.15 (−0.38, 0.67)
Short-term subgroup (n = 2)	No	Low	Low/acceptable	No	Very serious	Moderate	0.16 (−0.36, 0.67)
Tens vs. Placebo THI							
Post-treatment group (n = 3)	Serious	Moderate	low/acceptable	No	Very serious	Very Low	0.51 (0.1, 0.91)
Short-term subgroup (n = 2)	No	Low	low/acceptable	No	Very serious	Moderate	0.3 (−0.23, 0.83)
Tens vs. Placebo Vas Loudness							
Post-treatment group (n = 2)	No	Low	Low/acceptable	No	Very serious	Moderate	0.19 (−0.34, 0.71)
Short-term subgroup (n = 2)	No	Low	Low/acceptable	No	Very serious	Moderate	0.13 (−0.39, 0.65)
Tens vs. Placebo Vas Uncomfortable							
Post-treatment group (n = 2)	No	Low	Low/acceptable	No	Very serious	Moderate	0.07 (−0.45, 0−59)
Short-term subgroup (n = 2)	No	Low	Low/acceptable	No	Very serious	Moderate	0.16 (−0.36, 0.68)

## Data Availability

We analyzed our meta-analysis using an excel spreadsheet with the program Mix 2.0 Pro. We extracted the sample size, means, and standard deviations for each variable. When the trial reported standard error or non-parametric values (median or interquartile range), they were converted to standard deviations and parametric values as needed. If necessary, the mean scores and standard deviations were estimated from graphs. The between-groups mean differences of the trials were converted to effect sizes using a random-effects model. An effect size of 0.8 or greater was considered large, between 0.5 and 0.8 as moderate, and between 0.2 and 0.5 as small. *p*-values < 0.05 were considered statistically significant.
